# An Exploratory Survey of *Deqi* Sensation from the Views and Experiences of Chinese Patients and Acupuncturists

**DOI:** 10.1155/2013/430851

**Published:** 2013-11-21

**Authors:** Hong-Wen Yuan, Liang-Xiao Ma, Peng Zhang, Chi Lin, Dan-Dan Qi, Jing Li, Si-Yuan Xin, Ni-Juan Hu, Chun-Hua Li, Yu-Qi Liu, Jie Hao, Jie-Ping Xie, Hai Cui, Jiang Zhu

**Affiliations:** ^1^School of Acupuncture Moxibustion and Tuina, Beijing University of Chinese Medicine, Beijing 100029, China; ^2^School of Traditional Chinese Medicine, Capital Medical University, Beijing 100069, China; ^3^The Key Unit of Evaluation of Characteristic Acupuncture Therapy, State Administration of Traditional Chinese Medicine, Beijing 100029, China; ^4^Beijing Electric Power Hospital, Capital Medical University, Beijing 100073, China; ^5^Institute of Basic Research in Clinical Medicine, China Academy of Chinese Medical Science, Beijing 100700, China

## Abstract

*Deqi* sensation is believed to be important in clinical efficacy according to TCM theory. The measuring method of *Deqi* sensation has significant implications for the result of research trials. This study makes an investigation on acupuncture-experienced patients and expert acupuncturists in China and aims to find out the patient's needling sensations and acupuncturist's sensations which can be acceptable as descriptors of *Deqi* sensation, so as to provide foundation for more systematic and sensitive quantitative evaluation method of *Deqi* sensation. Results of this survey indicated that the *Deqi* sensation noted by both patient and acupuncturist is equally important to the treatment efficacy. It is found that there are some differences between the patients' real-life experience and the acupuncturists' expectations on patients' *Deqi* sensation. The “dull pain,” “aching,” “sore,” “numb,” “distended,” “heavy,” “electric,” “throbbing,” “warmness,” “coolness,” “spreading,” and “radiating” can be considered as the main manifestations of *Deqi* sensations. The acupuncturists believed that *Deqi* sensations were mainly “pulling,” “tight,” and “throbbing.” We suggest developing a questionnaire measuring the *Deqi* sensations which includes both the sensations of the patient and acupuncturist, and this would be very important and necessary for a better understanding of the relationship between *Deqi* sensation and acupuncture effects in future studies.

## 1. Introduction

*Deqi*, which first appeared in the “*Huang Di Nei Jing*,” refers to the reaction of movement of qi (vital energy) of the human body when acupuncture needles are inserted into acupoints. It is believed that *Deqi* was closely related to the treatment efficacy. In clinic, *Deqi* is mainly testified by needle sensation, which includes the patients' sensations and the acupuncturists' sensations. The patient's needle sensations mainly include sour, numb, swelling, heavy, pain, hot, cold, and the complex feeling and spreading based on these feelings; when the patients have those needling sensations, the acupuncturist can also feel heavy, tense, or needle stuck, or needle tremor [1–3]. The central mechanism of *Deqi* has been partly explored in some studies [4–9], but it does not have a well understanding. Preliminary findings suggest that *Deqi* may be an important factor of acupuncture analgesia [10–12]. Measuring the patients' *Deqi* sensation by using the international scale was originated from the end of the 1980s [13]. In recent years, in order to effectively improve the quality of acupuncture research on *Deqi*, quantitative measurement of *Deqi* attracts increasing attention. The representative scales include the MASS scale compiled by Kong et al. (the MGH Acupuncture Sensation Scale) [14] and the Southampton Needle Sensation Questionnaire written by White et al. [15]. In addition, some investigators developed scales in Korean [16]. Yu et al. have found that the Chinese version of MASS has good repeatability and internal consistency [17]. However, a standardized, valid, and reliable scale for quantitatively measuring the “*Deqi*” is still lacking.

In traditional acupuncture, the acupuncturists must pay attention to eliciting and controlling of *Deqi*. However, modern evaluation tools for *Deqi* and its related studies all ignore the acupuncturists' sensations. By taking a systematic investigation of the ancient and modern literature, we found that the current evaluation tools can be further improved based on well understanding of *Deqi*'s connotation. In order to develop a validated method to measure the* Deqi*, we performed a descriptive survey with the following three specific aims: (1) understanding Chinese patients' and acupuncturists' attitudes and beliefs on acupuncture sensations and *Deqi*, (2) exploring the influence factors of *Deqi* and the effect of *Deqi* on acupuncture treatment efficacy, (3) summarizing the patients' *Deqi* sensations reported by patients and senior acupuncturists, and (4) investigating the acupuncturist's perceptions on *Deqi.*

In China, the health care system includes both modern medicine and TCM. As acupuncture is very popular in China, *Deqi* sensations in an acupuncture treatment session are widely accepted by the majority of Chinese patients. Some patients even require obtaining the needling sensations or some other special acupuncture feelings on their own initiative [18]. The patients' attitude towards *Deqi *sensation was significant different between Chinese patients and those from the US [19]. So in this study, we investigated individual patients' experiences in an actual clinical setting and then consulted expert acupuncturists who have rich clinical experience. The survey is made by observing a homogeneous population undergoing the same style of TCM.

## 2. Methods

### 2.1. Survey Development

Prior to the formal investigation, a structured interview was setup through a literature review involving both ancient and modern, Chinese and English, and then the terms frequently used for describing the patients' and acupuncturists' perception on *Deqi *were collected. The Chinese literature includes the ancient books, modern acupuncture text books, and the published articles related to *Deqi*. The English literature includes the questionnaires of *Deqi*, or needle sensation, and scales used for measuring *Deqi* [13–16, 20, 21] and its related investigations [19, 22, 23]. The terms in the lists from the English literature were translated by two translators, respectively. We unified the terms that own the same meaning in Chinese but with different expressions in English, such as the terms “stretch,” “expansion,” “distention,” and “fullness,” usually appearing in the English literature, are all translated into “*Zhang*” (fullness/distention) in Chinese, and then we used the item “distention” in English in this paper.

The draft questionnaires were developed based on the interview with four doctoral candidates and four experts with senior professional titles from the School of Acupuncture-Moxibustion and Tuina, Beijing University of Chinese Medicine. These experts and doctoral candidates have rich experience in administrating acupuncture and being treated with acupuncture. Then, two expert acupuncturists and five patients completed the draft questionnaires. We also interviewed them regarding their opinions on using the questionnaires and modified the draft according to their comments. The major modifications for patient's questionnaire include making a detailed and more clear explanation to distinguish the similar items such as “pulling” and “tight,” “spreading” and “radiating.” In the options of the question “which terms can describe the *Deqi* sensations of the patients?” some additional interpretations have been added for the terms that may be puzzled to common patients. For example, “dull pain” is interpreted as “mild, moderate or minor pain without a strong inimical feeling,” “sharp pain” is interpreted as “a severe pain with a sense of injury,” “twinge” as “an unbearable pain with the highest intensity,” “pain” as “a pain unable to clarify its nature.” In the questionnaire for the acupuncturists, the questions of “spreading distance” and “the direction of spreading” of needling sensation were added.

Finally, the second presurvey was conducted through the interview on another six acupuncture-experienced undergraduates who were not majored in medicine. No modification was made in the first interview. After one week, we altered the order of the questions and conducted the interview for the same subjects again. The results were consistent with those obtained in the first interview.

### 2.2. Survey Content

There are two types of questionnaires, one is for the patients and the other is for the expert acupuncturists, which are completed by the patients and the experts, respectively.

The patient's questionnaire contained two parts and one open-ended question. Part 1 includes four single choice questions on the patients' perception of *Deqi*. Part 2 includes questions about the patient's sensations experienced during acupuncture administration. There are two phases in part 2. Phase 1 involves questions mainly asking about the patients' needling sensations. Twenty-six terms are separated into four categories: pain, temperature, sensation spreading, and others. One supplementary blank was at the end for subjects to describe sensations in their own words. Phase 2 includes three questions asking about the patients' emotions during acupuncture. An open-ended question is added at the end, “What is your impression on *Deqi*?” 

There are also two parts and one open-ended question in the questionnaire for the experts. It includes some questions in the questionnaire for the patients in order to get experts' perceptions on *Deqi* sensation of patients based on their clinical experience. Part 1 includes five multiple choice questions and three single choice questions regarding the understanding of *Deqi* by the experts. The questions in part 2 aimed to inquire expert's perceptions on needling sensations of the patient (the same questions in patient's questionnaire) and sensations felt by expert's needling hand. An open-ended question is added at the end, “What are your opinions on this questionnaire of *Deqi*?”

### 2.3. Administration

As the *Deqi* occurrence rate in Chinese clinical trials is 100% [24], it is hard to calculate the sample of the subjects. According to the suggestions of the experts, we performed a survey to 40 acupuncturists with senior professional titles and 40 patients, respectively. The survey was conducted in the Beijing TCM Hospital affiliated to Capital Medical University, Huguosi TCM Hospital, and Dongzhimen Hospital affiliated to Beijing University of Chinese Medicine in April 2012. All patients could complete the questionnaire by themselves. All questionnaires were completed by those patients and acupuncturists independently. All investigators have been trained before the investigation. The acupuncturists and patients were reminded that this questionnaire is related to the *Deqi *sensation only deriving from manual acupuncture with filiform needles (as there are varieties of needles in Chinese clinical treatments). The investigators tried to not disturb the subjects during completing the questionnaires and answered any questions of the subjects in a unified way. Only when the investigators found some missed questions or apparently contradictory options, they would take some necessary reminding. 

### 2.4. Statistical Analysis

The questionnaire data was independently collected by two persons. We made a statistics after verification and amending. Descriptive statistics were performed by using the computer and presented by percent (%). 

When inputting the data, we combined some terms which describe similar sensations, such as “warm” and “burnt,” considered to be a different degree of “warm,” are merged into “warm,” “cool” and “cold” are merged in to “cool.”

Types of patients' needling sensations perceived by patients and understood by acupuncturists were compared using chi-square analysis. Chi-square analysis and Fisher's exact test (when appropriate) were then performed to explore the effect of expressing by patients and acupuncturists on types of patients' acupuncture sensations. A level of *P* < 0.05 was determined to be of statistical significance, and all analyses were two-sided. Statistical analysis was performed using SPSS Statistics 17.0.

## 3. Results

### 3.1. Participants

A total of 40 patients participated in this survey; 70% of them were men, with an average age of 60.2 years old. Each of them had prior acupuncture experience, and 83% of the respondents had more than 10 times acupuncture treatment experiences. When needles are insert, 58% of the patients stated that they achieved needle sensations in each treatment, while the others always (22%) or occasionally (20%) have such feelings, and nobody stated that they had never had such feeling. Forty acupuncturists participated in this survey; 65% were women, with the average age of 48.5 and working time of 20.7 years. 

### 3.2. The Patients' Attitudes and Beliefs towards *Deqi*

In this study, nearly 70% of the patients never heard of “*Deqi*.” Among the patients who know about *Deqi*, four thought that *Deqi* is a sensation during acupuncture, one thought that *Deqi* meant the channels were unblocked, and three of them cannot explain the specific meaning. 85% of the patients expected to have the needle sensations upon the treatment, and only one did not expect to have needling sensation. About 90% of the patients thought needling sensation is closely related to the treatment efficacy, and others did not know their relationship. About 58% of the respondents said that the acupuncturist will ask them about their needle sensations during every acupuncture treatment session. Nearly 40% of the patients said that they were always asked by their acupuncturists. The patients in this study all have been asked about their needling sensation by their acupuncturists during the treatment, as shown in Table 1.

### 3.3. The Acupuncturists' Understanding about *Deqi*

In this study, 95% of the acupuncturists in China thought that “*Deqi*” should be elicited in the treatment, while others thought the treatment without needling sensation can also achieve the efficacy. Some experts said that needling sensation could not be elicited in every point due to limitation of treatment time. They said that the patients expected to get a stronger needling sensation, which is inconsistent with the results concluded from the questionnaires for the patients.

Most acupuncturists (90%) held that acupuncturists sensations felt by their needling hand and the needling sensations of the patients are the typical characteristics of *Deqi*. Compared to the acupuncturists, the patients are more easily to get needling sensation. In this study, all of the acupuncturists believed that the sensations of the acupuncturists can be a sign of *Deqi*.

About 73% of acupuncturists thought that *Deqi* sensation is related to the treatment efficacy, but only 38% of them thought that the symptoms can be relieved immediately. Most acupuncturists believed that the patients' sense of *Deqi* and the acupuncturists sensations are equally important to the treatment efficacy, as shown in Table 2.

Regarding the relationship between* Deqi* sensation and treatment efficacy, most acupuncturists thought that the efficacy is closely related to the existence of *Deqi* sensation (87%), the realization of qi spreading to the affected parts (47%), and the intensity of *Deqi* sensation (37%). In addition, one-third of the acupuncturists thought that the time for inducing *Deqi* sensation and the direction of sensation spreading may affect the efficacy. Only a few patients thought that the lasting time of *Deqi* sensation (11%) and what kind of sensation (8%) may affect the efficacy.

In addition, the acupuncturists held that the acupoint location (87.5%), point selection (82.5%), and the acupuncture manipulation (90%) are the three key elements in acupuncture. The physiques of the patients (80%), acupuncturist's needling skill (67.5%), needle depth (60%), the thickness of needle (57.5%), the type of disease (45%), the patient's emotional and psychological states (40%), acupoints selection (35%), needle inserting site (35%), needle retention time (22.5%), season while needling (25%), and time of acupuncture administration (15%) may all affect the efficacy.

### 3.4. The Patients' Perceptions of *Deqi* Sensation

The most common acupuncture sensations reported as experienced by patients were “distended” (72.5%), “aching” (65%), “sore” (60%), those “spreading” (60%), and, reported as considered by acupuncturists were “warmness” (92.5%), “sore” (87.5%), “aching” (85%), “distended” (85%), and “spreading” (82.5%), as shown in Table 3.

In this survey, 82.5% of the patients believed that *Deqi* sensation is not harmful in the treatment. However, nearly half of the patients considered that *Deqi* sensation is tolerable; none of them felt *Deqi* cannot be tolerated. Half of the respondents stated that *Deqi* sensation is comfortable. Some patients indicated that *Deqi* is just a series of sensations; they do not care whether it is comfortable or not, as shown in Table 4. 

### 3.5. The Relationship between Patients' Experience and Acupuncturists' Consideration of *Deqi* Sensation

In this study, we not only asked patients to select the terms on behalf of the needle sensation, but also invited some expert acupuncturists to filter them. It showed that there are no statistical differences among the 14 descriptors of the all 26 descriptors (*P* > 0.05). However, there are some differences between the patients' real-life experience and the acupuncturists' expectations among the others, as shown in Table 3. 

As long as the patients or acupuncturists select one option among the feelings in the same category, it will be considered to select this kind of feeling in statistics. After comparison, it is found that there is no significant difference between the patients and acupuncturists in selecting the pain related feeling (*P* > 0.05), which indicated that the majority of patients and acupuncturists believed that *Deqi* sensation is associated with pain.

There are significant differences between patients and acupuncturists in selecting the temperature feel and feeling spreading (*P* = 0.000), which suggested that patients actually feel the temperature changes and needling sensation spreading occurred less than the acupuncturists' expectation.

There are no differences between the “dull pain,” “distended,” “electric,” and “throbbing” between two groups of subjects (*P* > 0.05). All of these sensations are selected by more than half of the patients and acupuncturists, indicating that the feelings are thought to present signs of* Deqi* sensations.

Referring to the terms “sharp pain,” “twinge,” “throbbing pain,” “burning,” “penetrating,” “formicating,” their supporting rate has no statistical difference between the two groups (*P* > 0.05). Moreover, all are below 20%, indicating that these feelings mentioned above are not the typical *Deqi* sensations. One-third of the patients selected “pricking” significantly higher than the selection rate of acupuncturists (*P* = 0.005). 

### 3.6. The Acupuncturists' Experience of Needle Grasping Sensation

The most common sensations reported by acupuncturists were “pulling” (95%), which was described as “as a fish biting the hook” in ancient books, and “tight” (70%) as shown in Table 5.

## 4. Discussion

### 4.1. The Choice of the Respondents

One purpose of this survey is to filter the descriptors of the patients' *Deqi* sensation and the acupuncturists' sensations, so as to develop proper clinical evaluation questionnaires on *Deqi*. So it is expected to select acupuncture-experienced subjects, especially those patients who already recognized the *Deqi* sensation. This study is different from some previous studies which selected naive volunteers without any acupuncture experience as their subjects [13, 14, 22]. It has been reported that acupuncture not only benefits the chronic pain [25, 26], but also can be an effective adjunctive treatment for facilitating stroke rehabilitation [27, 28]; therefore, acupuncture is widely used for facilitating stroke rehabilitation in China [29, 30].

The long treatment course and large amount of stimulation in this treatment are particularly in consistent with the requirements of this survey. So in this study, we selected the patients from three outpatient acupuncture clinics of Beijing TCM hospitals for the survey. 70% of the respondents suffered from apoplexy and the others from facial paralysis, trauma, headache, low back pain, and gastrohelcoma. More than 80% of these patients have received more than 10 sessions of acupuncture treatment. They had more experiences in perceiving *Deqi* sensation and could rule out the needle sensations by accident. Compared with the respondents in experimental models, a survey based on these respondents may reflect the real phenomena of *Deqi* sensation in the real world clinical setting.

### 4.2. The Chinese Patients' and Acupuncturists' Attitudes of *Deqi*

The majority of the patients thought that needling sensations are related to the treatment efficacy and excepted to experience these sensations in the treatment. Nearly half of the patients thought *Deqi* is comfortable. Although some patients may have felt uncomfortable sometimes, they also thought it is not harmful. Our result is consistent with Mao's et al. result [31]. Regarding the open-ended questions, two patients said that eliciting the *Deqi* sensations indicated that the acupuncturist had good needling skills. 

In this survey, three quarters of the 40 experts thought *Deqi *sensation is closely related to the treatment efficacy. Although 25% of the acupuncturists were not sure about the relationship between *Deqi *sensation and treatment efficacy, they still excepted to elicit the *Deqi *sensation in clinical. It may be influenced by the acupuncture education and the demands of the patients. Only one acupuncturist thought *Deqi* sensation is not related to treatment efficacy. So it is obviously shown that the acupuncturists and patients, especially the patients who suffered more severe diseases, are eager to achieve *Deqi* sensation and highly expected its influence on efficacy.

### 4.3. The Patients' *Deqi* Sensation

This survey was a retrospective study, which filtered the terms to describe patient's *Deqi* sensations based on their memories. 92.5% of patients reported at least one pain related term. There are three terms used to describe the intensity of pain, “dull pain,” “sharp pain,” and “twinge.” Half of the patients chose dull pain, four of them chose sharp pain and dull pain, and one patient chose dull pain and twinge at the same time as the twinge occasionally occurred and it is difficult to distinguish them. Although most patients cannot distinguish pain and *Deqi* sensation, “dull pain” is widely accepted to be one of the* Deqi* sensations. In two terms of “deep pain” and “skin pain” associated with location of pain, more patients chose skin pain, indicating that the patients may have an incorrect understanding of *Deqi* sensation. *Deqi* sensation is the needle sensation when the needle was inserted into the acupoints with a certain depth, which pointed out that the pain caused by skin penetrating during needle inserting should be distinguished so as to avoid confusion of the skin pain and *Deqi* sensation. Among the twenty-six terms for needle sensations in this questionnaire, “fullness” was the most common sensation (72.5%), followed by the “aching” (65%) and “sore” (60%); approximately half of the patients selected “dull pain,” “numb,” “spreading,” which can be considered as the characteristics of *Deqi *sensations. 

In previous studies of filtering *Deqi* sensation terms, the investigated objects include both patients and acupuncturists [13, 14, 20, 22, 23]. This survey is quite different from previous studies. We filtered the terms of *Deqi *sensations by the patients and expert acupuncturists in the same hospital at the same time. After comparing, it is found that there exist some differences between the real-life experiences of patients and acupuncturists' expectation. 

It was found that there are no significant differences between the patients (92.5%) and acupuncturists (97.5%) in selecting the pain related terms (*P* > 0.05), suggesting that both of them believe that *Deqi *sensation is associated with pain. There are experimental studies that confirmed that the basic material for generating acupuncture needles sensation is mainly the pain receptors located in the acupoints with a depth [32, 33]. The dull pain, aching, sore, and distended sensation is consistent with its reflection in brain [34], so it showed that *Deqi *and pain cannot be completely separated in physiology.

There are much more acupuncturists (92.5%) selecting the temperature related sensation than the patients (25%) (*P* = 0.000). It suggests that patients actually rarely feel the temperature changes during acupuncture than the acupuncturist expected. A lot of heat- or cold-inducing needling manipulations recorded in acupuncture classics may be the reason that most acupuncturists tend to choose the warm feeling as the sign of* Deqi* (92.5%). Although the sense of cool is usually mentioned in the literature, but only 47.5% acupuncturists had successfully elicited a sense of cool and achieved a better efficacy at the same time. (This might be caused by the types of diseases, and the cool sensation is more difficult to be elicited.) As there is only 1 patient who had experienced such feeling, significant differences existed between the two groups (*P* = 0.000). However, we believed that the difference was just due to the small sample in this survey.

Although it has a significant statistical difference (*P* = 0.000) between the patients (72.5%) and acupuncturists (100%) in selecting the sensation spreading (*P* = 0.000), the choosing rates of the two groups of people are relatively high, which means that propagated sensation along channel is also one of the typical manifestations of *Deqi *sensations.

Among the twenty-six terms, “dull pain,” “sore,” “aching,” “numb,” “distended,” “electric,” “warmness,” and “spreading,” their supporting rate reached 50% or above, and these feelings are the common feelings appearing in *Deqi* sensation. However, the supporting rate of “sharp pain,” “twinge,” “throbbing pain,” “burning,” “penetrating,” and “formicating” were below 20%, indicating that these are not the typical signs of* Deqi *sensations.

About 1/3 of the patients chose the “sting” significantly higher than the choice rate of the acupuncturists (*P* = 0.005). The terms “pricking” and “stinging” occurring rate is above 60% among the young females, which is nearly one time higher than the 32.5% in this study [22]. It obviously showed that pricking always appeared accompanied with *Deqi* sensations, but most acupuncturists thought that pricking is only a feeling during needle inserting and does not belong to *Deqi *sensation [13, 34–37]. Recently, it has been found that pricking pain is not commonly regarded as *Deqi* sensation, so the acupuncturists should avoid pricking pain to the patients.

The choose rates of “coolness,” “heavy,” and “radiating” among the acupuncturists are all above 50%, while they are below 20% among the patients. The “coolness” discussed above is one type of *Deqi *sensations as mentioned in ancient books. However, "coolness" is hard to be elicited in the clinical due to the type of disease, which may be the main reason that there was a significant difference between the patients and acupuncturists. In two hundred cases of retrospective investigation, 57.5% of patients achieved a heavy sense [31], and another two hundred and twenty seven cases in a survey conducted in UK stated that the heavy sense choosing rate is 20% [15], which confirmed that heavy is a possible sense of *Deqi* sensation. There are “radiating” related terms in the literature of Vincent et al. [13] and Park et al. [22]. A survey of Korean women with a similar sample size reported that “radiating” appearance rate is 76.3% [23], while it disappeared in the MASS and SNSQ list. In this study, in order to facilitate patients' selections, we added explanations for the terms radiating and spreading. Radiating: needles sensation spread without fixed directions; spreading: needles sensation spread with a fixed direction. 

### 4.4. The Acupuncturist's Needle Grasping Sensation of *Deqi*

In recent *Deqi* studies, more attention is paid to the patients' needle sensations than the acupuncturists sensations [13–16]. However, according to traditional theory the acupuncturists' sensations are more important in eliciting and controlling *Deqi*. Therefore, this survey adds more terms of the description of the acupuncturists' sensations. In this study, most expert acupuncturists believed that *Deqi* sensation is mainly manifested by “pulling” and “tight”, which is the same as described in ancient Chinese medicine. A few of them thought “throbbing” is beneficial to the treatment of cerebrovascular disease caused by motor dysfunction and other diseases, which is regarded as one kind of *Deqi* sensation. However, most acupuncturists do not agree with “opposable” mentioned in some Chinese pieces of literature (5%).

### 4.5. Limits

Some acupuncture sensations had to be translated from English into Chinese, and we have to add certain additional interpretations for better understanding by Chinese patients. Then, all the terms had to be retranslated to English again, whereby the delicate meaning of some of the items may not have been fully conveyed in its Chinese equivalent. The sample size of this study was not big enough, and the scope of this survey is narrow. 

All of the objects in this study were acupuncture-experienced patients, and more are men. However, this investigation can still reflect the general recognitions of *Deqi *sensations among the Chinese patients and acupuncturists. In the future, we would make an investigation with a larger sample and analyze the relation of the frequency of occurrence of different *Deqi* sensation with curative effect.

## 5. Conclusion

Results of this survey indicated that acupuncturists and patients, especially the patients in serious condition, are eager to get *Deqi* sensations and highly expected to get better efficacy due to stronger *Deqi* sensation. 

Different from previous studies, this survey filtered the terms of *Deqi* sensations by the patients and experts in the same hospital at the same time. After comparing, it is found that there exist some differences between the real-life experiences of patients and acupuncturists' expectation. It is found that pain related feeling and sensation spreading feeling are the major manifestations of *Deqi* sensations. However, the patients actually feel the temperature changes, and acupuncture conduction occurred less than the acupuncturists' expectation.

The “dull pain,” “sore,” “aching,” “numb,” “distended,” “electric,” “throbbing,” “warmness,” “spreading,” “coolness,” “heavy,” and “radiating” can be considered as the main manifestations of *Deqi* sensations, while “sharp pain,” “twinge,” “pricking,” “throbbing pain,” “burning,” “penetrating,” “pinch,” and “formicating” may not be the typical signs of *Deqi* sensations. The acupuncturists' *Deqi* sensations are mainly described as “pulling,” “tight,” and “throbbing”.

## Figures and Tables

**Table 1:** Patients' attitudes and beliefs about acupuncture sensation (*Deqi*).

Questions	Attitudes and beliefs	Number/%
Have you heard of* Deqi*?	Have heard	12/30%
	Never	28/70%

Do you expect to have needling sensation during acupuncture administration?	Expected	34/85%
	Dispensable	5/12.5%
	Try to avoid	1/2.5%
	Hate	0/0%

Have your acupuncturists asked you about your needling sensations?	Every time	23/57.5%
	Often	16/40%
	Seldom	1/2.5%
	Never	0/0%

Do you think needling sensation is close to the acupuncture treatment efficacy?	Yes	36/90%
	No	0/0%
	Not sure	4/10%

**Table 2:** Acupuncturists' understanding about *Deqi*.

Questions	Understanding	Number/%
Do you expect to get *Deqi *sensation in the acupuncture treatment?	Yes	38/95%
	No	1/2.5%
	Do not care	1/2.5%

Do your patients expect to get *Deqi* sensation in the acupuncture treatment?	Expected	29/72.5%
	Dispensable	9/22.5%
	Try to avoid	0/0%
	Very fear	0/0%
	Not sure	1/2.5%

What do you think are the signs of *Deqi*?	Patients needle sensation	36/90%
	Acupuncturists experiencing sensations	39/97.5%
	Muscle throbbing	20/50%
	Immediate relief	15/37.5%

Which are more likely to occur, the patients needle sensations or the acupuncturists sensations?	Acupuncturists experiencing sensations	11/27.5%
	Patients needle sensations	20/50%
	At the same time	9/22.5%

Do you think the *Deqi* sensation is related to the clinical efficacy?	Has	29/72.5%
	No	1/2.5%
	Not sure	10/25%

Between the acupuncturists sensations and the patients needle sensation, which do you think is more relevant to its efficacy?	Acupuncturists experiencing sensations	8/20%
	Patients needle sensations	13/35%
	With same significance	18/45%
	Not related	1/2.5%

**Table 3:** Types of patients' *Deqi *sensation.

Sensation	Experienced by patients %	Considered by acupuncturists %	*P* value
Related to pain	92.5%	97.5%	0.608
Dull pain	47.5%	60%	0.262
Shape pain	10%	2.5%	0.356
Twinge	5%	2.5%	1.000
Deep pain	17.5%	30%	1.000
Superficial pain	27.5%	20%	0.284
Pricking	32.5%	7.5%	0.005
Throbbing pain	12.5%	10%	1.000
Aching	65%	85%	0.039
Pain	10%	0	0.124
Related to temperature	25%	92.5%	0.000
Warmness	45%	92.5%	0.000
Burning	2.5%	12.5%	0.203
Coolness	2.5%	47.5%	0.000
Other feelings			
Penetrating	17.5%	10%	0.330
Sore	60%	87.5%	0.005
Numb	47.5%	75%	0.012
Distended	72.5%	85%	0.172
Heavy	12.5%	77.5%	0.000
Pressure	2.5%	30%	0.001
Pinch	2.5%	7.5%	0.608
Pulling	17.5%	37.5%	0.045
Tight	7.5%	30%	0.010
Electric	42.5%	50%	0.501
Formicating	10%	22.5%	0.130
Throbbing	42.5%	45%	0.491
Related to spreading	72.5%	100%	0.000
Spreading	60%	82.5%	0.026
Radiating	17.5%	57.5%	0.000

**Table 4:** Patients' affections about* Deqi* sensation.

Questions	Affections	Number/%
What do you think of needle sensation/*Deqi* in the treatment?	Harmful	1/2.5%
	No harm	33/82.5%
	Unknown	6/15%

What do you think of *Deqi* sensation?	Negligible	4/10%
	Gentle	21/52.5%
	Strong	7/17.5%
	Unbearable	0/0%
	Sometimes strong, sometimes weak	7/17.5%

What is your usual perception of *Deqi* sensation?	Comfortable	20/50%
	Uncomfortable	0/0%
	Does not matter	20/50%

**Table 5:** Types of acupuncturists experiencing sensation.

Sensation	Experienced by acupuncturists %
Pulling/heavy/like a fish biting the hook	38/95%
Tight	28/70%
Throbbing	6/15%
Opposable	1/2.5%

## References

[1] Cheng X (1987). *Chinese Acupuncture and Moxibustion*.

[2] Yuan YX, Ren J, Huang L (1997). *Chinese-English Dictionary of Traditional Chinese Medicine*.

[3] Shi XM (2007). *Text of Acupuncture and Moxibustion*.

[4] Bossy J, Prat D, Sambuc P (1984). Sensory potentials evoked by stimulation of the Jing points at the hand. *Acupuncture and Electro-Therapeutics Research*.

[5] Wang KM, Yao SM, Xian YL, Hou ZL (1985). A study on the receptive field of acupoints and the relationship between characteristics of needling sensation and groups of afferent fibres. *Scientia Sinica B*.

[6] Kagitani F, Uchida S, Hotta H, Aikawa Y (2005). Manual acupuncture needle stimulation of the rat hindlimb activates groups I, II, III and IV single afferent nerve fibers in the dorsal spinal roots. *Japanese Journal of Physiology*.

[7] Campbell A (2006). Role of C tactile fibres in touch and emotion—clinical and research relevance to acupuncture. *Acupuncture in Medicine*.

[8] Kong J, Gollub RL, Webb JM, Kong J-T, Vangel MG, Kwong K (2007). Test-retest study of fMRI signal change evoked by electroacupuncture stimulation. *NeuroImage*.

[9] Huang Y, Zeng TJ, Zhang GF (2012). Activated and deactivated functional brain areas in the *Deqi* state A functional MRI study. *Neural Regeneration Research*.

[10] Cao X (2002). Scientific bases of acupuncture analgesia. *Acupuncture and Electro-Therapeutics Research*.

[11] Ai L, Dai JP, Zhao BX (2004). Investigation of analgesic mechanism of acupuncture: a fMRI study. *Chinese Journal of Medical Imaging Technology*.

[12] Kim GH, Yeom M, Yin CS (2010). Acupuncture manipulation enhances anti-nociceptive effect on formalin-induced pain in rats. *Neurological Research*.

[13] Vincent CA, Richardson PH, Black JJ, Pither CE (1989). The significance of needle placement site in acupuncture. *Journal of Psychosomatic Research*.

[14] Kong J, Gollub R, Huang T (2007). Acupuncture De Qi, from qualitative history to quantitative measurement. *Journal of Alternative and Complementary Medicine*.

[15] White P, Bishop F, Hardy H (2008). Southampton needle sensation questionnaire: development and validation of a measure to gauge acupuncture needle sensation. *Journal of Alternative and Complementary Medicine*.

[16] Kim Y, Park J, Lee H, Bang H, Park H-J (2008). Content validity of an acupuncture sensation questionnaire. *Journal of Alternative and Complementary Medicine*.

[17] Yu DT, Jones AY, Pang MY (2012). Development and validation of the Chinese version of the Massachusetts General Hospital Acupuncture Sensation Scale: an exploratory and methodological study. *Acupuncture in Medicine*.

[18] Huang T, Wang RH (2010). An investigation report about the present situation of acupuncture Deqi. *Medicine & Philosophy*.

[19] Hui KKKS, Sporko TN, Vangel MG, Li M, Fang J, Lao L (2011). Perception of *Deqi* by Chinese and American acupuncturists: a pilot survey. *Chinese Medicine*.

[20] Kong J, Fufa DT, Gerber AJ (2005). Psychophysical outcomes from a randomized pilot study of manual, electro, and sham acupuncture treatment on experimentally induced thermal pain. *Journal of Pain*.

[21] Yin CS, Park J, Lee J-Y (2009). Acupuncture perception (*Deqi*) varies over different points and by gender with two distinct distribution patterns of dullness and pain. *Journal of Sensory Studies*.

[22] Park H, Park J, Lee H, Lee H (2002). Does *Deqi* (needle sensation) exist?. *American Journal of Chinese Medicine*.

[23] Park J, Park H, Lee H, Lim S, Ahn K, Lee H (2005). *Deqi* sensation between the acupuncture-experienced and the Naïve: a Korean study II. *American Journal of Chinese Medicine*.

[24] Xiong J, Yuan Q, Zhang LJ (2010). Preliminary observation of acupuncture *Deqi* in clinical. *Journal of Traditional Chinese Medicine*.

[25] Vickers AJ, Cronin AM (2012). Acupuncture for chronic pain: individual patient data meta-analysis. *Archives of Internal Medicine*.

[26] Coeytaux RR, Park JJ (2013). Acupuncture research in the era of comparative effectiveness research. *Archives of Internal Medicine*.

[27] NIH Consensus Statement.

[28] Shiflett SC (2007). Does acupuncture work for stroke rehabilitation: what do recent clinical trials really show?. *Topics in Stroke Rehabilitation*.

[29] Zhang S, Li N, Liu M (2009). Use of acupuncture for stroke in China. *Acupuncture in Medicine*.

[30] Zhao XF, Du Y, Liu PG (2012). Acupuncture for stroke: evidence of effectiveness, safety, and cost from systematic reviews. *Topics in Stroke Rehabilitation*.

[31] Mao JJ, Farrar JT, Armstrong K, Donahue A, Ngo J, Bowman MA (2007). De qi: Chinese acupuncture patients’ experiences and beliefs regarding acupuncture needling sensation—an exploratory survey. *Acupuncture in Medicine*.

[32] Wang K, Liu J (1989). Needling sensation receptor of an acupoint supplied by the median nerve—studies of their electro-physiological characteristics. *American Journal of Chinese Medicine*.

[33] Dong QS, Wang XQ, Zhang RT (2007). Discuss the material basis of manual acupuncture from the relationship between manual acupuncture sensation and sensory function in acupoint. *Journal of Sichuan of Traditional Chinese Medicine*.

[34] Hui KKS, Nixon EE, Vangel MG (2007). Characterization of the *Deqi* response in acupuncture. *BMC Complementary and Alternative Medicine*.

[35] Hui KKS, Marina O, Claunch JD (2009). Acupuncture mobilizes the brain’s default mode and its anti-correlated network in healthy subjects. *Brain Research*.

[36] Hui KKS, Marina O, Liu J, Rosen BR, Kwong KK (2010). Acupuncture, the limbic system, and the anticorrelated networks of the brain. *Autonomic Neuroscience*.

[37] Asghar AU, Green G, Lythgoe MF, Lewith G, MacPherson H (2010). Acupuncture needling sensation: the neural correlates of *Deqi* using fMRI. *Brain Research*.

